# Morphine Poisoning

**Published:** 1898-10

**Authors:** 


MORPHINE POISONING.—Dr. F. A. Remley, a valued subscriber to the TEXAS MEDICAL JOURNAL, reports the case of a little girl—age not stated—who swallowed two grains or morphine (eight one-quarter grain pills). He gave her atropia for the first twelve hours, and permanganate of potash for the next twelve hours. Dr. Remley says: "Thirty minutes after ingesting the permanganate of potash she would rally and breathe for one or two hours; then would quit again. Upon repeating the dose she would recover consciousness and remain conscious for a while, when respiration would almost cease again." She recovered.

The doctor adds: "I am satisfied that the permanganate potash is our best remedy."

